# Improving single nucleotide polymorphisms genotyping accuracy for dihydropyrimidine dehydrogenase testing in pharmacogenetics

**DOI:** 10.37349/etat.2024.00223

**Published:** 2024-04-24

**Authors:** Annalaura Montella, Sueva Cantalupo, Giuseppe D’Alterio, Vincenzo Damiano, Achille Iolascon, Mario Capasso

**Affiliations:** University of Rome La Sapienza, Italy; ^1^Department of Molecular Medicine and Medical Biotechnology, University of Naples Federico II, 80131 Naples, Italy; ^2^CEINGE Biotecnologie Avanzate Franco Salvatore, 80145 Naples, Italy; ^3^European School of Molecular Medicine, Università Degli Studi di Milano, 20122 Milan, Italy; ^4^Medical Oncology, Integrated Activity Department of Onco-Hematological Diseases, Pathological Anatomy and Rheumatic Diseases, AOU Federico II, 80131 Naples, Italy

**Keywords:** Pharmacogenetics, dihydropyrimidine dehydrogenase, genotyping

## Abstract

Fluoropyrimidines, crucial in cancer treatment, often cause toxicity concerns even at standard doses. Toxic accumulation of fluoropyrimidine metabolites, culminating in adverse effects, can stem from impaired dihydropyrimidine dehydrogenase (DPYD) enzymatic function. Emerging evidence underscores the role of single nucleotide polymorphisms (SNPs) in *DPYD* gene, capable of inducing DPYD activity deficiency. Consequently, *DPYD* genotyping’s importance is on the rise in clinical practice before initiating fluoropyrimidine treatment. Although polymerase chain reaction (PCR) followed by Sanger sequencing (SS; PCR-SS) is a prevalent method for *DPYD* genotyping, it may encounter limitations. In this context, there is reported a case in which a routine PCR-SS approach for genotyping *DPYD* SNP rs55886062 failed in a proband of African descent. The Clinical Pharmacogenetics Implementation Consortium (CPIC) categorizes the guanine (G) allele of this SNP as non-functional. The enforcement of whole genome sequencing (WGS) approach led to the identification of two adenine (A) insertions near the PCR primers annealing regions in the proband, responsible for a sequence frameshift and a genotyping error for rs55886062. These SNPs (rs145228578, 1-97981199-T-TA and rs141050810, 1-97981622-G-GA) were extremely rare in non-Finnish Europeans (0.05%) but prevalent in African populations (16%). Although limited evidence was available for these SNPs, they were catalogued as benign variants in public databases. Notably, these two SNPs exhibited a high linkage disequilibrium [LD; squared correlation coefficient (*R*^2^) = 0.98]. These findings highlighted the importance to consider the prevalence of genetic variants within diverse ethnic populations when designing primers and probes for SNP genotyping in pharmacogenetic testing. This preventive measure is essential to avoid sequence frameshifts or primer misalignments arising from SNP occurrences in the genome, which can compromise PCR-SS and lead to genotyping failures. Furthermore, this case highlights the significance of exploring alternative genotyping approaches, like WGS, when confronted with challenges associated with conventional techniques.

## Introduction

Fluoropyrimidines, including 5-fluorouracil and its pre-prodrug capecitabine, are frequently used for the treatment of pancreatic, colorectal, breast, gastric, head and neck cancers [1]. Although these drugs represent safe and effective chemotherapeutics, they frequently lead to the development of toxicity even at standard doses, recording up to 30% risk of severe adverse events up to date [2].

One of the causes of this toxicity is the deficiency of the enzyme dihydropyrimidine dehydrogenase (DPYD), encoded by *DPYD* gene and involved in fluoropyrimidines catabolism. An impairment of DPYD enzymatic function causes a toxic accumulation of fluoropyrimidine metabolites with consequent implication in terms of adverse events [3–6]. Recent evidence ascribes to germline pathogenic variants in the *DPYD* gene a crucial role in the development of DPYD deficiency, leading to a reduction in DPYD activity [7–9]. As a result, it has become increasingly common to perform *DPYD* genotyping prior to initiating treatment with fluoropyrimidines. This practice is aimed to the essential purpose of mitigating adverse events and minimizing treatment-related toxicity. By identifying individuals with these genetic variants in advance, healthcare providers can tailor treatment plans and dosages to enhance both the safety and efficacy of fluoropyrimidine-based therapies.

In light of these advancements, numerous medical agencies worldwide have issued guidelines recommending upfront *DPYD* testing for patients scheduled to receive fluoropyrimidine-based drugs [10–13]. The Clinical Pharmacogenetics Implementation Consortium (CPIC) has played a prominent role in this regard, setting guidelines for *DPYD* genotyping when determining fluoropyrimidine dosing. These guidelines emphasize four specific single nucleotide polymorphisms (SNPs) within the *DPYD* gene: c.1905 + 1guanine (G) > adenine (A; rs3918290), c.1679thymidine (T) > G (rs55886062), c.2846A > T (rs67376798), and c.1236G > A (rs56038477), with the latter serving as the tag SNP for haplotype B3 (HapB3), in association with rs75017182 c.1129 – 5923cytosine (C) > G [11]. These SNPs have been singled out due to their significant prevalence in the population and their established impact on enzyme function and the risk of treatment-related toxicity.

In particular, c.1905 + 1G > A (rs3918290) represents the first *DPYD* variant to be associated with a 50% reduced function (for heterozygous patients) or non-activity (for homozygous ones) of the enzyme, increasing the risk of toxicity after fluoropyrimidines treatment [14]. Later, c.2846A > T (rs67376798), c.1679T > G (rs55886062) and c.1236G > A (rs56038477) were identified in correlation with a 3.02, 4.4 and 1.59-fold relative risk of severe fluoropyrimidines-induced toxicity, respectively [15]. Another SNP associated with the decrease of DPYD enzymatic activity is the missense variant c.2194G > A (rs1801160), which is also correlated to an approximately 2.4- and 1.9-fold increased risk of haematological toxicity and neutropenia [16]. Considering all these evidences, *DPYD* genotyping is becoming more and more popular in clinical practice before chemotherapy treatment.

The gold standard method to perform pharmacogenetic tests, including genotyping, is polymerase chain reaction (PCR) followed by Sanger sequencing (SS). While this approach may be more expensive than other genotyping technologies, it is the method of choice for genotyping cancer patients for *DPYD* SNPs due to its reliability and precision. This approach employs primer pairs designed to target specific regions of the *DPYD* gene, encompassing exon 11, 13, 14, 18, 22, and intron 10, to investigate variants like rs56038477, rs55886062, rs1801160, rs67376798, rs3918290, and rs75017182.

However, in a recent case involving a proband of African ethnic origin, genotyping of *DPYD* rs55886062 using PCR-SS encountered difficulties, resulting in an electropherogram sequence frameshift. To overcome this issue, whole genome sequencing (WGS) was employed. The implementation of WGS led to successfully genotyped rs55886062 and also revealed the presence of two additional SNPs, 1-97981199-T-TA and 1-97981622-G-GA, in the patient. These new identified SNPs were responsible for the genotyping failure encountered with the traditional method. This case highlights the importance of considering alternative genotyping approaches, such as WGS, when facing challenges with standard techniques. Moreover, it points out also the importance to consider the prevalence of genetic variants within diverse ethnic populations when designing primers and probes for SNP genotyping in pharmacogenetic testing, such as PCR followed by the SS method.

## Case report

A 48-year-old male of Nigerian origin has been diagnosed with adenocarcinoma (grade 2) in the right colon. The tumor has spread to one lymph node (pT3N1 M1c), and genetic analysis reveals the presence of the p.Gly12Asp (c.35G > A) mutation in codon 12 of exon 2 of the *KRAS* gene within the tumor tissue.

*DPYD* pharmacogenetic test was required to treat the patient with advanced first-line chemotherapy according to the FOLFOX scheme (oxaliplatin in combination with fluorouracil and folic acid) with the addition of Bevacizumab of which he had already completed six cycles.

### Results

**Figure 1:**
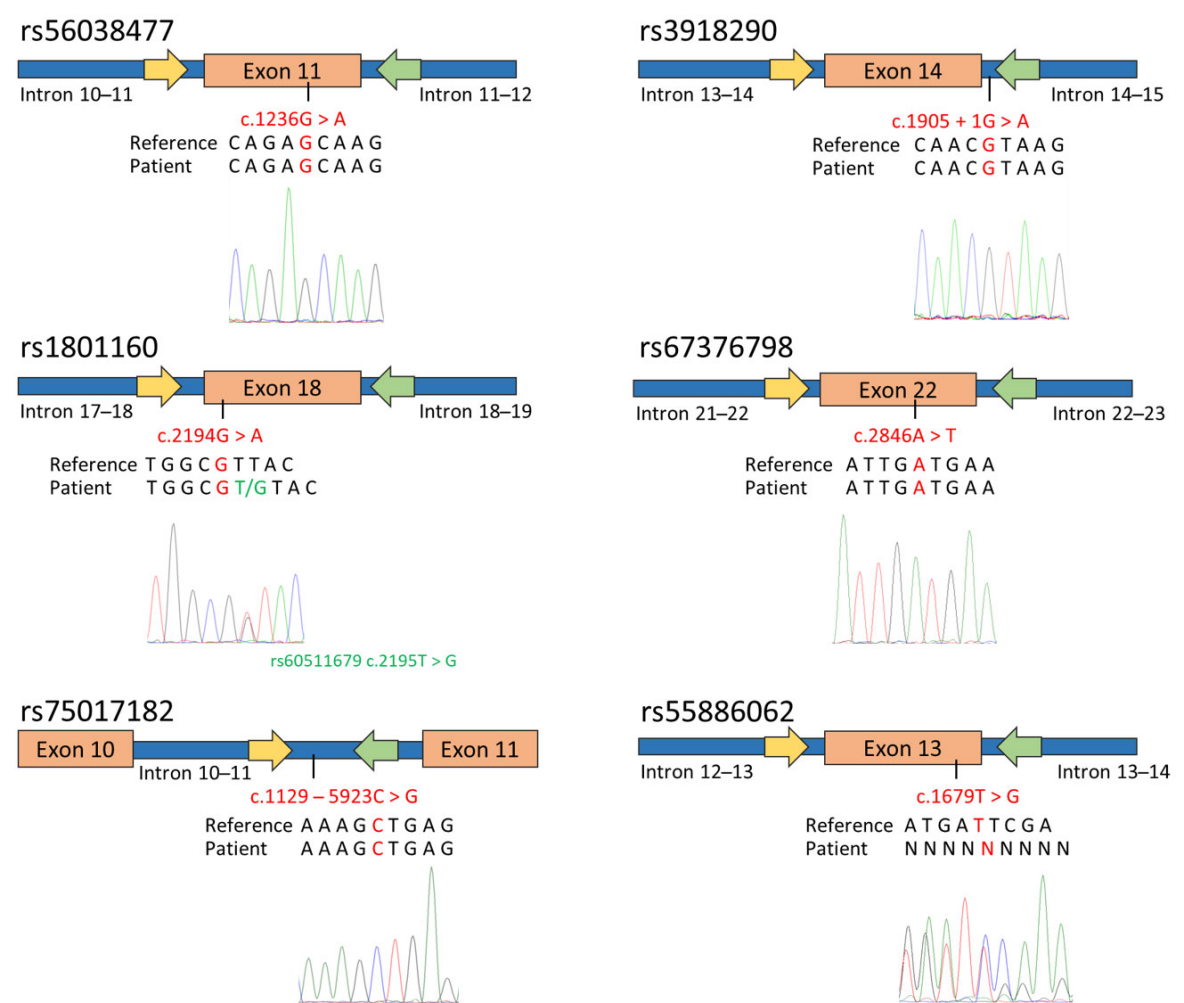
Genotyping of *DPYD* SNPs associated with toxicity and enzymatic activity reduction in the patient diagnosed with adenocarcinoma. The figure schematically presents the outcomes of SS for exon 11, 13, 14 (including a portion of intron 14–15), 18, 22, and intron 10. The yellow arrows indicate the forward primers used for PCR and sequencing, while the green arrows represent the reverse primers. Below the schematic representation, are reported the GRCh37/hg19 reference genome, the patient’s sequence, and the electropherograms obtained through SS using the forward primer. The investigated SNPs are highlighted in red

To predict potential toxicity in an adenocarcinoma patient prior to fluorouracil treatment, the genotyping of specific *DPYD* SNPs, including rs56038477, rs55886062, rs1801160, rs67376798, rs3918290, and rs75017182 was performed. Using already reported primers pair [17], PCR-SS revealed that the patient carried the most common genotypes for rs56038477, rs1801160, rs67376798, rs3918290, and rs75017182 (Figure 1). However, the patient exhibited heterozygosity for rs61622928 (c.12184G > A) and rs60511679 (c.2195T > G), which are not associated with a reduction in DPYD enzymatic activity, as documented in the PharmGKB database [18, 19].

Concerning the SNP rs55886062, a reading frameshift was observed in the electropherogram using both the forward and reverse primers (Figure 1). In order to indirectly determine the patient’s genotype for rs55886062, the LDlink program was used to assess the linkage among rs55886062, the examined *DPYD* SNPs and the two additional SNPs identified in the patient. Since no evidence of LD between rs55886062 and these SNPs (*R*^2^ = 0.00) were found, the employment of WGS was necessary to genotype rs55886062.

**Figure 2:**
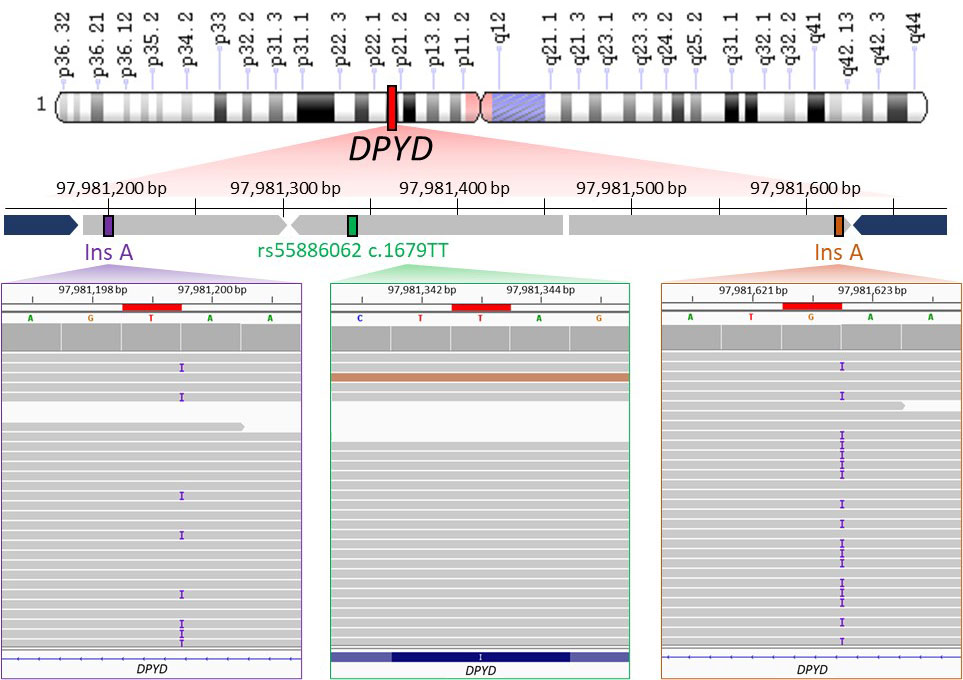
WGS data visualization. The upper part of image shows the schematic representation of WGS results about the genomic region (chr1: 97981161–97981656) containing rs55886062 (chr1: 97981343). Chromosome short and long arms are reported as “p” and “q” letter in the scheme, respectively. WGS identified the presence of T/T genotype for rs55886062 (depicted in green) and an A insertion downstream the primer of the forward primer (rs145228578 or 1-97981199-T-TA, shown in purple) and upstream of the annealing region of reverse primer (rs141050810 or 1-97981622-G-GA, shown in orange). The primers used for PCR are represented in blue. In the lower part of figure, binary alignment and map (BAM) files containing WGS data from the proband are loaded into the Integrative Genomics Viewer (IGV). Noteworthy findings are highlighted in red boxes, including rs55886062 (in a green box), rs145228578 or 1-97981199-T-TA (in a purple box) and rs141050810 or 1-97981622-G-GA (in an orange box)

Therefore, WGS results confirmed that the patient carried the homozygous T allele for rs55886062 (Figure 2, in green) and highlighted presence of one-base insertion A located downstream the forward primer (Figure 2, in violet) and another insertion upstream the annealing region of the reverse primer (Figure 2, in orange). Both insertions were found in close proximity of A repeats.

**Figure 3:**
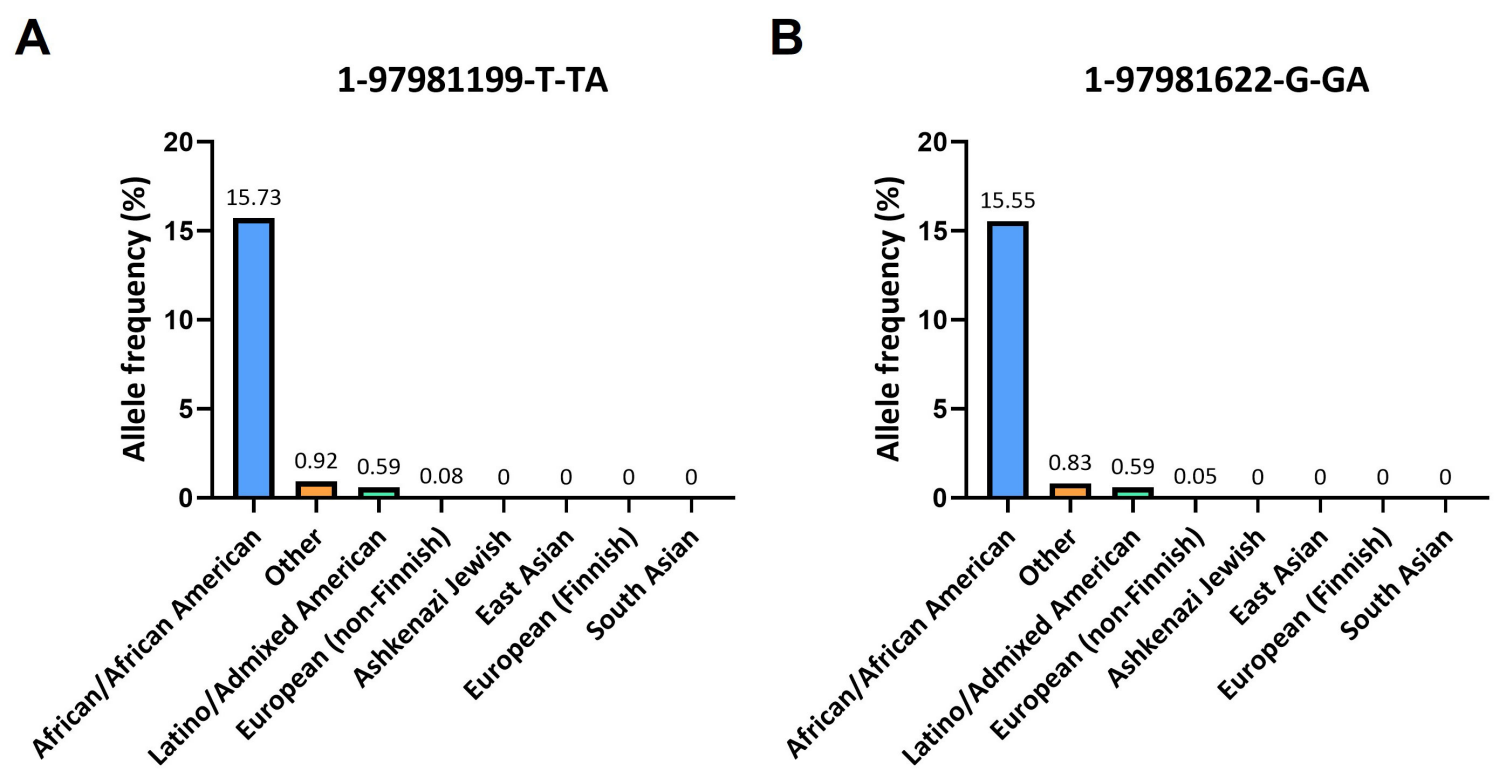
Frequencies of 1-97981199-T-TA and 1-97981622-G-GA across various ethnic populations. (A) Histogram displays the population frequencies of 1-97981199-T-TA obtained from the gnomAD v2.1.1 database; (B) the population frequencies of 1-97981622-G-GA taken from the same database are reported

In the gnomAD database, the A insertion near the alignment site of the forward primer corresponded to the variant 1-97981199-T-TA (rs145228578), while the other A insertion upstream the annealing region of the reverse primer corresponded to the variant 1-97981622-G-GA (rs141050810). It is worth noting that these genetic variants were found to be quite rare among non-Finnish Europeans (0.05%) but more prevalent in Africans (16%; Figure 3).

This frequency is also observed within certain Nigerian ethnicities: for the Esan and Yoruba in Ibadan, Nigeria, the frequencies of the variant 1-97981199-T-TA are 16.2% and 17.1% respectively, while for the variant 1-97981622-G-GA are 16.2% and 16.7% respectively (Table 1).

**Table 1:** Allele frequencies of 1-97981199-T-TA and 1-97981622-G-GA across African ancestry populations

**Population**	1-97981199-T-TA*	1-97981622-G-GA*
African Caribbean in Barbados	18.2%	18.2%
African Ancestry in Southwest US	18.0%	18.0%
Esan in Nigeria	16.2%	16.2%
Gambian in Western Division, The Gambia	26.5%	26.1%
Luhya in Webuye, Kenya	15.7%	15.7%
Mende in Sierra Leone	17.6%	17.6%
Yoruba in Ibadan, Nigeria	17.1%	16.7%

* Allele frequencies (%) obtained from 1000 Genomes Project

In both ClinVar and Franklin databases, 1-97981199-T-TA and 1-97981622-G-GA were reported as benign variants. Moreover, the enquiry of LDlink tool highlighted that these two SNPs were in LD (*R*^2^ = 0.99).

**Figure 4:**
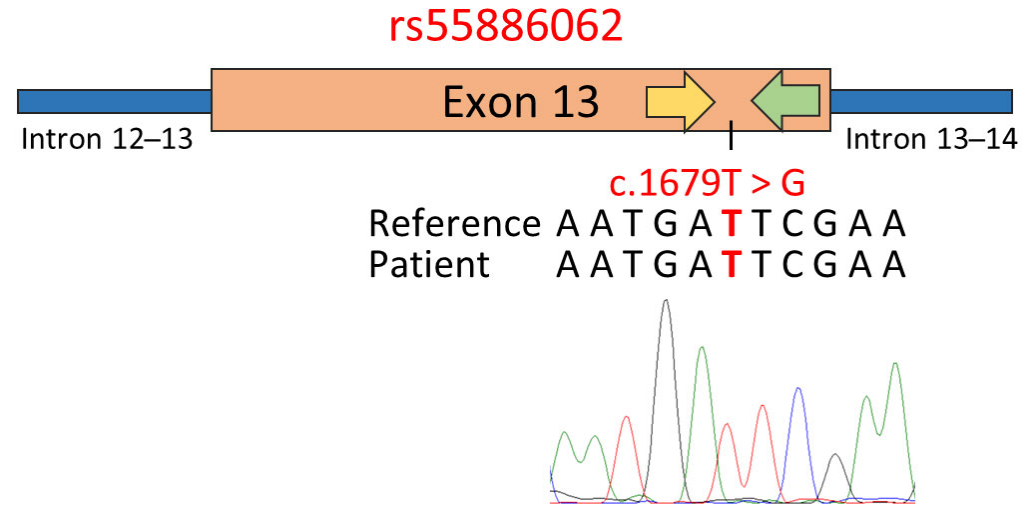
Genotyping of rs55886062 using primer pairs designed in close proximity to the SNP site. The schematic representation displays the results of SS, employing a primer pair positioned within the region flanked by the primers previously used to amplify exon 13 of the *DPYD* gene. In the figure, the yellow arrow represents the forward primer used for PCR and sequencing, while the green arrow represents the reverse primer. Below the representation, the GRCh37/hg19 reference genome, the patient’s sequence, and the electropherogram obtained via SS with the forward primer are reported. The investigated SNPs are highlighted in red

Altogether these findings suggested that the presence of 1-97981199-T-TA and 1-97981622-G-GA near the PCR primer annealing regions led to the electropherogram frameshift and the SS failure. In order to address this issue, an alternative primer pair within the region flanked by the primers previously used for amplifying *DPYD* exon 13 was developed. Subsequent sequencing of this amplified region successfully verified the patient’s genotype for rs55886062, as shown in Figure 4.

### Material and methods

#### DNA extraction

Patient venous blood was collected into dipotassium-ethylenediaminetetraacetic acid (K2-EDTA) tube (Becton, Dickinson Vacutainer) and processed by using Wizard^®^ Genomic DNA Purification Kit (Promega), according to manufacturer instruction. DNA purity was checked using the NanoPhotometer^®^ spectrophotometer (IMPLEN) and its quality was assessed on 0.8% agarose gel.

#### PCR and SS

To routinely perform genotyping for *DPYD* SNPs strongly linked to toxicity following fluoropyrimidines treatment (specifically, rs56038477, rs55886062, rs1801160, rs67376798, rs3918290, and rs75017182), a PCR on exon 11, 13, 14 (including a segment of intron 14), 18, 22, and intron 10 was carried out using primer pairs previously documented in the literature [17]. All PCRs were performed using KAPA2G Robust HotStart ReadyMix PCR Kit (Kapa Biosystems) at the cycle conditions of 95°C for 3 min, followed by 35 cycles of 95°C for 30 s, 60°C for 45 s, and 72°C for 45 s with a final elongation step of 72°C for 1 min. The amplification of exon 13 inside the region flanked by 1-97981199-T-TA and 1-97981622-G-GA was performed using the following primers: 5’-tgtaaaacgacggccagtAGAAATGGCCGGATTGAAGT-3’ and 5’-caggaaacagctatgaccAAGTTTTGGTGAGGGCAAAACC-3’ (M13 sequence is highlighted in lower case). After performing PCR with KAPA2G Robust HotStart ReadyMix PCR Kit at the cycle conditions of 95°C for 3 min, followed by 35 cycles of 95°C for 15 s, 65°C for 15 s, and 72°C for 15 s with a final elongation step of 72°C for 1 min, PCR purification and SS were performed by DNA lab facility at the institute “CEINGE Biotecnologie Avanzate Franco Salvatore”.

#### WGS

WGS of the patient was performed on an Illumina HiSeq1500 platform at 35× depth. The paired-end sequencing produced 150 bp long reads which were aligned to the GRCh37/hg19 reference genome using Burrows-Wheeler Alignment with maximal exact matches (BWA-MEM2) tool (doi:10.1109/IPDPS.2019.00041). PCR duplicates were marked and removed using the MarkDuplicates tool of the genome analysis toolkit (GATK) suite [20]. SNPs and insertions and deletions (INDELs) were detected using the HaplotypeCaller program of GATK suite [20]. The resulting variant call format (VCF) files were annotated with ANNOtate VARiation (ANNOVAR) [21].

#### Evaluation of SNPs frequencies and pathogenicity

GnomAD v2.1.1 (https://gnomad.broadinstitute.org/) and 1000 Genomes Project Phase 3 (Ensembl, https://grch37.ensembl.org/) databases were used to evaluate SNP frequencies in different populations. To evaluate SNPs pathogenicity, ClinVar (https://www.ncbi.nlm.nih.gov/clinvar/) and Franklin (https://franklin.genoox.com/) were consulted. SNP linkage was analysed using the LDlink tools (https://ldlink.nih.gov/).

## Discussion

Fluoropyrimidines constitute common medications for treating cancers with 2 million of treated patients annually [22–24]. Indeed, they remain the most effective drugs often used alone or in combination with other medications for treatment of colorectal (1.8 million), gastric (1 million), and pancreatic (460 thousand) cancers [25]. However, fluoropyrimidines may cause significant toxicities and adverse side effects such as nausea/vomiting, diarrhea, mucositis, alopecia, myelosuppression, heart toxicity, hand-foot syndrome (HFS), leukopenia and neutropenia [26]. Nowadays, the treatment-related mortality rate is 0.2–1.0% [27].

Underlying the severe adverse effects from treatment with fluoropyrimidines there are four *DPYD* SNPs (rs3918290, rs67376798, rs55886062 and rs56038477) considered the most clinically relevant and with statistically significant association with severe toxicity [2]. For this reason, several international agencies and consortia released recommendations on *DPYD* testing prior to treatment with fluoropyrimidines [10–13]. The current *DPYD* guideline released by CPIC recommends to reduce the dose of fluoropyrimidines by 25–50%, from the full standard dose, in individuals with a DPYD activity score of 1.5 [11]. Moreover, a recent prospective study provides evidence for genotype-guided dosing of decreased function alleles/variants, supporting a recommendation for a 50% dose reduction in heterozygous carriers of the decreased function variants rs67376798 or rs75017182 [2].

In the current scenario, the *DPYD* genotyping test is becoming increasingly essential before initiating fluoropyrimidines treatment. In fact, oncologists are now more frequently requesting *DPYD* genotyping for cancer patients, leading genetic laboratories to dedicate greater efforts towards enhancing the efficiency and accuracy of these genetic tests to deliver rapid and reliable results. Among the several technologies employed for *DPYD* genotyping, PCR-SS stands out as the most reliable method, despite its relatively high cost. However, it is worth noting that a significant portion of the primers used in genetic screening targets intronic regions, which are under less selective pressure compared to coding ones. Therefore, these intron-based primers tend to accumulate a greater number of genetic alterations.

Using primer pairs previously documented in the literature [17], *DPYD* genotyping was performed in an adenocarcinoma patient of African descent through PCR-SS. During this process, a challenge in rs55886062 genotyping was encountered, which was ultimately resolved using a WGS approach. WGS revealed the presence of two nucleotide insertions, 1-97981199-T-TA and 1-97981622-G-GA, near the annealing regions of the primers employed for amplifying *DPYD* exon 13. Specifically, 1-97981199-T-TA was situated in intron 12–13, while 1-97981622-G-GA resided in intron 13–14 of the *DPYD* gene. Both of these variants were identified as benign intronic variants in ClinVar and Franklin. Notably, these two variants were found to be more prevalent in the African population, which matches the patient’s ethnic background. Additionally, it is worth mentioning that 1-97981199-T-TA and 1-97981622-G-GA were observed to be in LD.

The presence of these two genetic variants led to a frameshift sequence in the electropherogram, making the detection of rs55886062 unfeasible. To address this limitation, the usage of a secondary primer pair within exon 13, designed to exclude the regions where the patient’s SNPs (1-97981199-T-TA and 1-97981622-G-GA) are located, was needed.

This case study underscores the critical importance of considering alternative genotyping approaches, such as WGS, when conventional techniques represent challenges. It highlights the need for flexibility in genotyping methods to accommodate unexpected complexities in an individual’s genetic makeup, especially in the context of pharmacogenetic testing. Additionally, this case serves as a valuable reminder of the significance of taking into account the diversity of genetic variants within different ethnic populations when designing primers and probes for SNP genotyping in pharmacogenetic testing. Genetic diversity among various ethnic groups can significantly impact the accuracy and reliability of genotyping methods. Hence, when developing genotyping assays for pharmacogenetics, it is crucial to consider the genetic variability and adapt the approach accordingly to ensure comprehensive and accurate results, particularly in a multicultural and diverse patient population.

## Abbreviations

A: adenine
C: cytosine
DPYD: dihydropyrimidine dehydrogenase
G: guanine
LD: linkage disequilibrium
PCR: polymerase chain reaction
PCR-SS: polymerase chain reaction followed by Sanger sequencing
SNPs: single nucleotide polymorphisms
SS: Sanger sequencing
T: thymidine
WGS: whole genome sequencing

## Declarations

### Copyright

© The Author(s) 2024.
